# Influenza and Enteric Fever

**Published:** 1900-11-24

**Authors:** 


### The Hospital

A Journal of

The Medical Sciences and Hospital Administration.

Vol. XXIX.—No. 739.] Edited by Sir Henry Burdett, K.C.B., and Solomon C. Smith, M.D., M.R.C.P. [November 24, 1900

### Influenza and Enteric Fever.

One of the best ascertained facts in pathology is that the various infectious fevers to which the human frame is liable are caused by the growth within the body of various organisms to which, for convenience, we give the generic title of microbes. These, being implanted in the body from without, grow and elaborate certain poisonous products, each after its kind; and according to the manner in which these products—these toxins—affect the economy, and the manner in which the body reacts to the embarrassing new conditions in which it finds itself, so is the disease produced, be it enteric, or influenza, or some other kind. Hence, although, taking the whole course of the disease, each specific germ implants its own specific character upon the malady, a very large proportion of the symptoms met with are not the direct product of the disease germ or its toxins, but are the outcome of the body's efforts to resist the invasion to which it is being subjected, and as there are only certain lines along which resistance can be effected, it is not to be wondered at that, if we put on one side such directly toxic signs as rashes, there is a general similarity between the early symptoms of many febrile diseases. Thus it happens that, in forming a diagnosis, dependence has often to be placed upon the progress and succession of the symptoms rather than upon the picture presented by their grouping at any given moment. Enteric fever and influenza, for example, are diseases which differ greatly in their course. Yet they are not infrequently mistaken for each other in their early stages. This was well illustrated in an outbreak of enteric fever which occurred in Clifton (Davies in *Medico-Chirurgical Transactions*, vol. lxxxi.) a year or two ago, a considerable number of the earlier cases having been thought to be suffering from influenza, and their true nature having only being discovered by the energy of the medical officer of health in bringing Widal's test to bear upon the problem. We are again reminded of this matter by the reports which have come to hand in regard to the illness of the Tsar, which, after being at first regarded as influenza, was later on discovered to be enteric or typhoid fever, or, as it is often called on the Continent, typhus abdominalis. Enteric fever may easily be confused with other diseases, especially during the first week. In patients who have suffered from tuberculous disease in any form, it may be a matter of great doubt for several days whether one has to do with enteric fever or with some secondary tuberculous affection—tuberculous meningitis, or acute general miliary tuberculosis. In regard to influenza, it is well recognised that the gastro-intestinal form of this disease may closely simulate enteric fever. According to Dreschfeld, we may have a roseolar rash, tympanites, gurgling, a markedly enlarged spleen, and profuse diarrhœa, while the pyrexia, as in enteric fever, may last some weeks. Moreover, as a still further complication, one must not forget that influenza and enteric fever may occur together, so that a case may appear to be a typical one of influenza, and indeed may form one of a group of such cases, and yet after the subsidence of the pyrexia due to the influenza a rise of temperature may again take place, and the case may run through all the symptoms of enteric fever, showing that there has been a double infliction. As to the question of diagnosis between influenza and enteric fever, it is to be noted that although in influenza there may be in some rare cases a roseolar rash, this tends to be more widely distributed than the spots of enteric, and does not come out in successive crops, as so generally occurs in the latter disease. Then the gurgling in the abdomen is not confined to the right iliac fossa, but is more generally distributed, as in ordinary diarrhœa. The course of the fever tells much if one has it from the beginning. The sudden onset of the pyrexia in influenza, sometimes running the temperature up to 103° Fahr. or 104° Fahr. in the first twenty-four hours, would of course go to negative enteric; as also would the absolutely sudden onset of the other symptoms, if one could be sure that the apparent date of onset was the real beginning of the disease. Unfortunately, a sufficiently large number of cases of enteric fever pass through the early days of the disease without actual breakdown to make it sometimes difficult to feel sure that the given dates of onset are correct. What may appear to be the onset of an influenza may really be the breakdown of a case of ambulant enteric fever. This same uncertainty as to dates, and especially the fact that the early days of enteric fever have often gone by before the patient comes under observation, may deprive us of the very great assistance which the course of the temperature, as described by Wunderlich, gives when it is observed. Where there is cough the discovery of influenza bacilli in the expectoration might clear up the diagnosis. The more specific diagnostic signs of enteric fever are often unfortunately not very definite at the very time when their help is most required. The diazo reaction is often absent during the first week, and even when present is by no means peculiar to this disease. If typhoid bacilli can be discovered in the urine, that would be clear evidence; but this sign is often absent in the first week. Horton Smith says that the bacilli may make their first appearance in the urine at any period of the disease, but that the condition is rare before the third week. Widal's reaction, again, is valuable when it occurs in a distinctive degree; but it does not usually appear until the second week, although it may in some cases be met with between the fifth and eighth days. What is sufficiently clear is that in the early days of enteric fever it may be most difficult to arrive at anything like a positive diagnosis, and that in attempting to distinguish it from certain forms of influenza our dependence must be placed upon the course and development of the symptoms rather than upon individual signs.

